# Design and development of novel MRI compatible zirconium- ruthenium alloys with ultralow magnetic susceptibility

**DOI:** 10.1038/srep24414

**Published:** 2016-04-19

**Authors:** H.F. Li, F.Y. Zhou, L. Li, Y.F. Zheng

**Affiliations:** 1 Department of Materials Science and Engineering, College of Engineering, Peking University, Beijing 100871, China; 2 Center for Biomedical Materials and Engineering, Harbin Engineering University, Harbin 150001, China

## Abstract

In the present study, novel MRI compatible zirconium-ruthenium alloys with ultralow magnetic susceptibility were developed for biomedical and therapeutic devices under MRI diagnostics environments. The results demonstrated that alloying with ruthenium into pure zirconium would significantly increase the strength and hardness properties. The corrosion resistance of zirconium-ruthenium alloys increased significantly. High cell viability could be found and healthy cell morphology observed when culturing MG 63 osteoblast-like cells and L-929 fibroblast cells with zirconium-ruthenium alloys, whereas the hemolysis rates of zirconium-ruthenium alloys are <1%, much lower than 5%, the safe value for biomaterials according to ISO 10993-4 standard. Compared with conventional biomedical 316L stainless steel, Co–Cr alloys and Ti-based alloys, the magnetic susceptibilities of the zirconium-ruthenium alloys (1.25 × 10^−6^ cm^3^·g^−1^–1.29 × 10^−6^ cm^3^·g^−1^ for zirconium-ruthenium alloys) are ultralow, about one-third that of Ti-based alloys (Ti–6Al–4V, ~3.5 × 10^−6^ cm^3^·g^−1^, CP Ti and Ti–6Al–7Nb, ~3.0 × 10^−6^ cm^3^·g^−1^), and one-sixth that of Co–Cr alloys (Co–Cr–Mo, ~7.7 × 10^−6^ cm^3^·g^−1^). Among the Zr–Ru alloy series, Zr–1Ru demonstrates enhanced mechanical properties, excellent corrosion resistance and cell viability with lowest magnetic susceptibility, and thus is the optimal Zr–Ru alloy system as therapeutic devices under MRI diagnostics environments.

Biomedical metallic alloys are the remarkably important members in the field of biomedical materials and therapeutic devices. It has been reported that more than 70% of biomedical implants and devices are made of metallic biomaterials^1^. The most commonly used metallic biomaterials for the past decades include stainless steels (316L SS, 304 SS), cobalt (Co)–chromium (Cr) alloys, and titanium (Ti) and its alloys. However, these conventional metallic biomaterials with high magnetic susceptibility have been reported to have adverse effect on the Magnetic Resonance Imaging (MRI) and diagnostics as they would become magnetized in the intense magnetic field of the MRI and diagnostics instruments, causing heat generation in the biomedical materials and therapeutic devices, displacement of the biomedical materials and therapeutic devices, and artifacts on the MRI and diagnostics^2^,^3^,^4^,^5^. Such artifacts can distort the authentic bioimaging and diagnostics of the human organs and tissues around the implant, thus preventing the exact diagnosis. The areas affected by the artifacts are related to the high magnetic susceptibility of the implant and would decrease with a decrease in the magnetic susceptibility^6^,^7^. In consideration of the MRI compatibility, materials and devices with an ultralow magnetic susceptibility are required for surgery and diagnostics performed under MRI.

Ruthenium (Ru) belongs to the platinum group and has ultralow magnetic susceptibility, similar to the alloying elements Nb, Mo, Rh, Pd and Ag, Ru belongs to the 4*d* transition metals in the Periodic Table of Elements^8^. Ru is extremely promising for potential biomedical applications, as they are extremely biocompatible, exhibiting low ionic cytotoxicity *in vitro*, excellent biocompatibility *in vivo*, no evidence of mutagenicity or carcinogenicity, a good resistance to corrosion, and osteocompatibility equaling or exceeding that of the conventional pure Ti and Ti-based biomedical materials^9^,^10^,^11^,^12^,^13^,^14^. Ru is known to enhance the corrosion resistance of biomedical Ti-based alloys by several orders of magnitude with even a 0.1% addition Ru and has been widely reported to be incorporated into the biomedical Ti-based alloys^14^,^15^. Previous studies demonstrated that adding Ru into Zr–based alloys can further enhance the microhardness and wear resistance^16^. According to the Zr–Ru binary phase diagram^17^, which indicates that the solubility of Ru in the Zr matrix is 12 wt.% (β phase) and 1 wt.% (α phase) respectively. And when exceeding the solubility, RuZr intermetallic would separate out.

In the present study, Zr–Ru alloys with various Ru alloying element content were designed and fabricated for novel biomedical Zr alloys with ultralow magnetic susceptibility, enhanced mechanical properties, improved corrosion resistance, excellent biocompatibility and MRI compatibility. In addition, the as-cast alloys were further undergone cold deformation and annealing treatment in order to further modify the mechanical properties and corrosion resistance of the Zr–Ru alloys.

## Results

### Microstructures properties of Zr–Ru alloys

**Figure 1:**
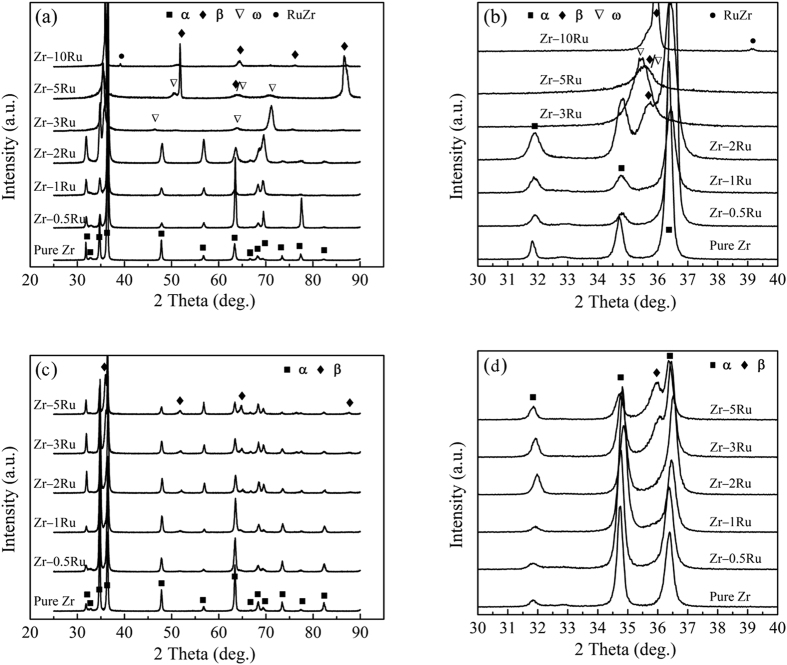
XRD patterns of as-cast (**a,b**) and annealed (**c,d**) Zr–Ru alloys, (**b,d**) are the corresponding enlarged views of (**a,c**) in the range of 30°~40°.

Figure 1 shows the XRD patterns of as-cast (Fig. 1(a,b)) and annealed (Fig. 1(c,d)) Zr–Ru alloys. For the as-cast Zr–Ru binary alloys, it can be seen from Fig. 1 that similar to pure zirconium, the Zr–0.5Ru, Zr–1Ru and Zr–2Ru alloys exhibited a single hexagonal close-packed structure (α phase). With the increase of the Ru content, the ω and β phases appeared in the XRD patterns of Zr–3Ru, Zr–5Ru and Zr–10Ru alloys. In addition, the RuZr phase was observed in Zr–10Ru alloy. After being cold worked to 50% thickness reduction and subsequently annealed at 600 °C, recrystallization occurred in the Zr–Ru alloys, as indicated by the reappearance of the α phase and the disappearance of the metastable ω phase in the Zr–3Ru and Zr–5Ru alloys.

The optical metallographic graphs of the as-cast and annealed Zr–Ru alloys are shown in the Supplementary Information S1. The as-cast pure Zr and Zr–Ru alloys showed coarse dendrite grain with the diameter larger than 500 μm, as illustrated in Supplementary Information S1A. On the other hand, the as-annealed pure Zr and Zr–Ru alloys demonstrated much smaller grain sizes, less than 50 μm, as revealed by Supplementary Information S1B.

### Mechanical properties of Zr–Ru alloys

**Figure 2:**
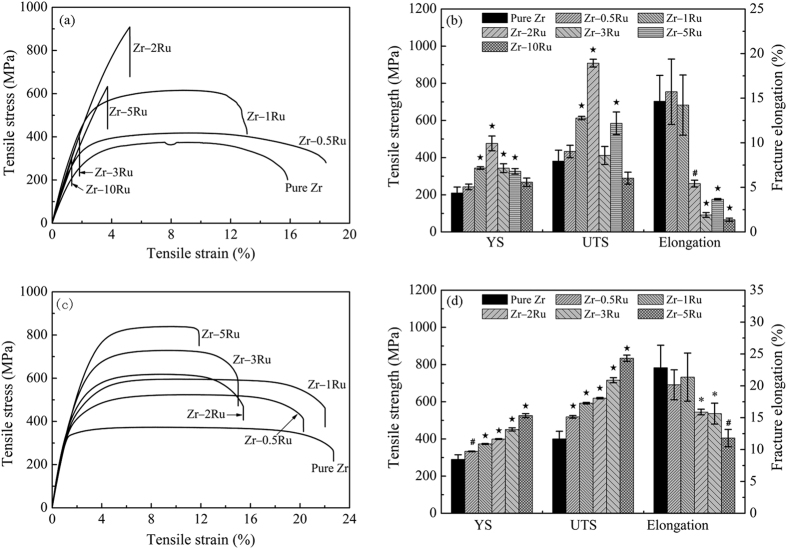
The tensile stress-strain curves (**a,c**) and tensile mechanical property data (**b,d**) of as-cast (**a,b**) and annealed (**c,d**) pure Zr and Zr–Ru alloys (*indicating p < 0.05, ^#^indicating p < 0.01 and ^⋆^indicating p < 0.001 when comparing with pure Zr).

The tensile stress-strain curves (a, c) and tensile mechanical property data (b, d) of as-cast (a, b) and annealed (c, d) pure Zr and Zr–Ru alloys are shown in Fig. 2. It can be seen that both the Yield Strength (YS) and the Ultimate Tensile Strength (UTS) of the Zr–Ru alloys are higher than that of pure Zr, except for the as-cast Zr–10Ru alloy, of which the UTS is slightly lower than that of the as-cast pure Zr, as the as-cast Zr–10Ru alloy exhibited brittle fracture during tensile test with the lowest elongation of 1.4%. After recrystallization annealing, pure Zr and Zr–Ru alloys exhibited higher ductility, as their average elongation at fracture exceeded 10%.

**Figure 3:**
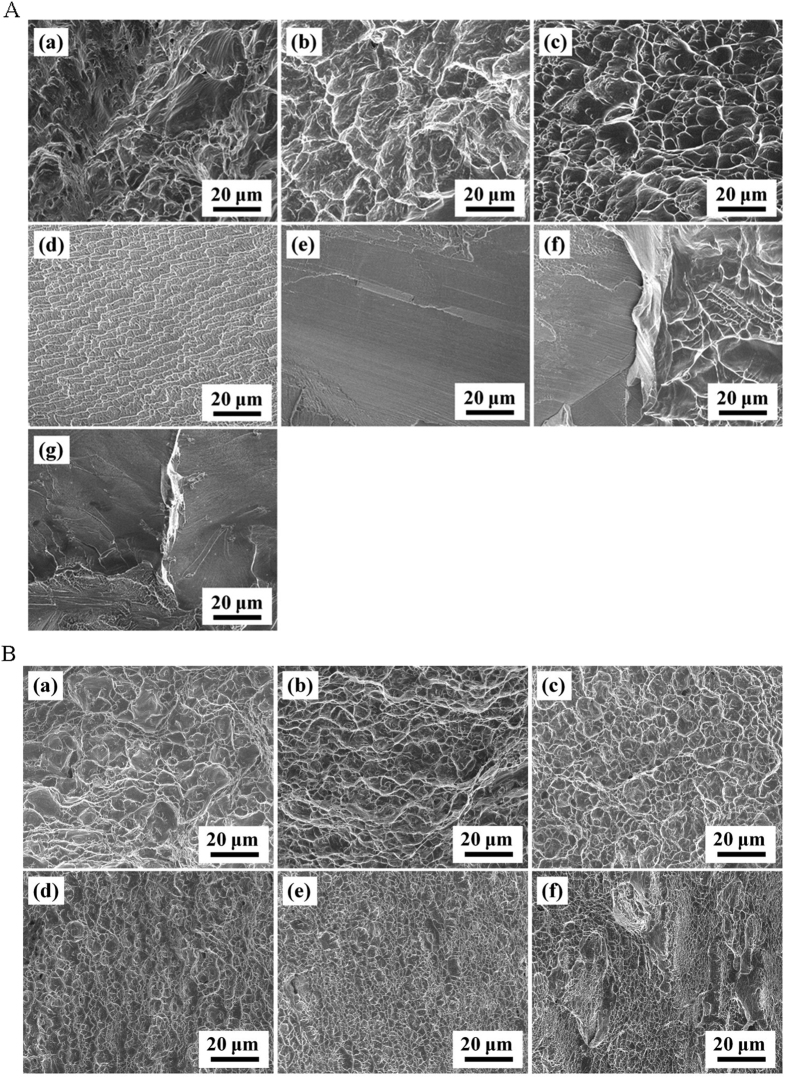
Fracture micrograph of as-cast (Fig. 3A) and annealed (Fig. 3B) pure Zr and Zr–Ru alloys: (**a**) pure Zr, (**b**) Zr–0.5Ru, (**c**) Zr–1Ru, (**d**) Zr–2Ru, (**e**) Zr–3Ru, (**f**) Zr–5Ru and (**g**) Zr–10Ru alloy samples.

Figure 3 showed the fracture micrograph of as-cast (Fig. 3A) and annealed (Fig. 3B) pure Zr and Zr–Ru alloys. It is obvious that the fracture morphology characteristics consistent with the results of tensile experiments. For the as-cast groups, the fracture morphology of pure Zr, Zr–0.5Ru and Zr–1Ru exhibited typical toughness fracture, with many dimple patterns being observed. The dimples of the Zr–2Ru alloy are very small and shallow, indicating the decreased toughness. With the further increase of the content of Ru, the Zr–3Ru, Zr–5Ru and Zr–10Ru alloy exhibited typical cleavage fracture, indicating their relatively poor toughness. After annealing, all the Zr–Ru alloys exhibited typical toughness fracture, with many dimple patterns observed.

**Figure 4:**
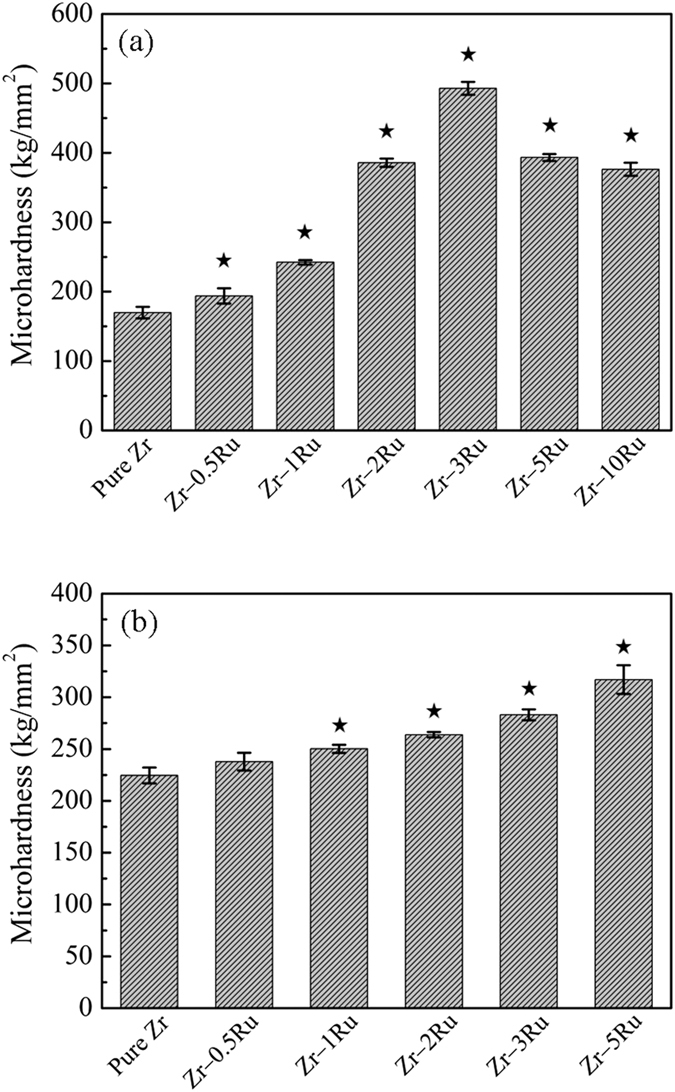
Microhardness of as-cast (**a**) and annealed (**b**) pure Zr and Zr–Ru alloys (^⋆^indicating p < 0.001 when comparing with pure Zr).

Figure 4 showed the microhardness of as-cast (a) and annealed (b) pure Zr and Zr–Ru alloys. It can be seen that after adding the alloying element Ru, the microhardness of Zr was enhanced obviously. Both the trends of the as-cast (a) and annealed (b) alloys are consistent with the corresponding tensile test results. For the as-cast alloys, the trend is in the parabola type, with the Zr–3Ru alloy having the highest microhardness(493 kg/mm^2^). For the as-annealed alloys, the trend is a monotonicity rise, which results in Zr–5Ru alloy having the highest microhardnesss.

### Electrochemical corrosion behaviors of Zr–Ru alloys

**Figure 5:**
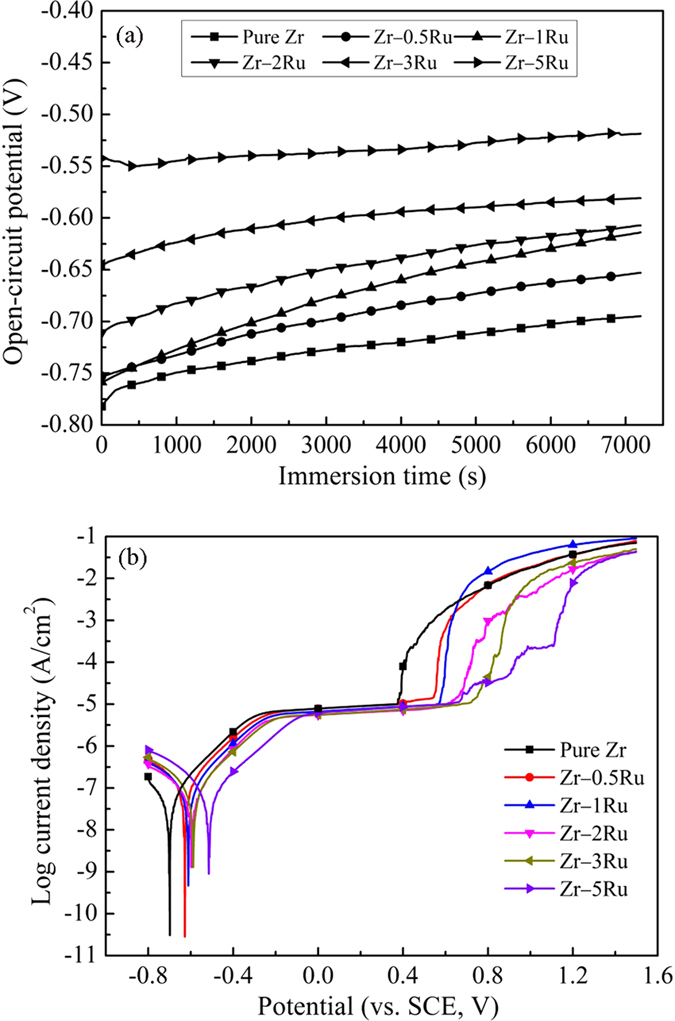
Electrochemical test of annealed pure Zr and Zr–Ru alloys in Hank’s solution, (**a**) OCP curves, (**b**) Potentiodynamic polarization curves.

Figure 5 demonstrated the electrochemical test of annealed pure Zr and Zr–Ru alloys in Hank’s solution. According to the open circuit potential (OCP) curves (Fig. 5(a)) and the potentiodynamic polarization curves (Fig. 5(b)), the following parameters including OCP, the corrosion potential (*E*_*corr*_), the corrosion current density (*i*_*corr*_) and the breakdown potential (*E*_*tran*_) can be calculated, as listed in Supplementary Information S2. As shown in Fig. 5(a), the OCPs change slowly towards noble potentials and reach relatively stable values for pure Zr and Zr–Ru alloys during 2 h exposure in Hank’s solution. The continuous increase of OCP implies that the passive film spontaneously formed on the metallic surface. By comparing the OCP values (Supplementary Information S2), after alloying with Ru, these alloys show increased OCPs compared to pure Zr, which suggested that Ru additions made the spontaneous passive film more stable thermodynamically, thus providing these Zr–Ru alloys higher corrosion resistance compared to pure Zr. As shown in Fig. 5(b), a passive region was observed on the anodic branch of the polarization curve before the transpassivation occurrence, indicating the thickening and growth of passive film (oxide). It was evident that current plateaus of Zr–Ru alloys were uniformly lower than that of pure Zr, which suggested that the alloying increased the passivity of pure Zr, showing a better protection against dissolving. At more positive potentials, the passive films broke down and the current densities increased rapidly. It can be found in Supplementary Information S2 that all experimental Zr–Ru alloys exhibited lower corrosion current densities compared to pure Zr, which further suggested that Ru alloy additions improved the corrosion resistance of pure Zr. Furthermore, the breakdown potentials (*E*_*tran*_) of Zr–Ru alloys are much higher than that of pure Zr, further indicating the enhanced pitting corrosion resistance by adding the Ru alloying element.

The element distributions of Zr–Ru alloys before and after corrosion test were detected by XPS analysis and the results are listed in Supplementary Information S3 and S4. The XPS analysis demonstrated that major components on the corroded surface of Zr–Ru alloys are ZrO_2_ and RuO_2_, and with the increasing of the Ru content, the content of RuO_2_ increased. It can be observed that with the increasing ratio of the Ru-oxide to Zr-oxide, the corrosion resistance increased.

### Evaluation of *in-vitro* biocompatibility of Zr–Ru alloys

**Figure 6:**
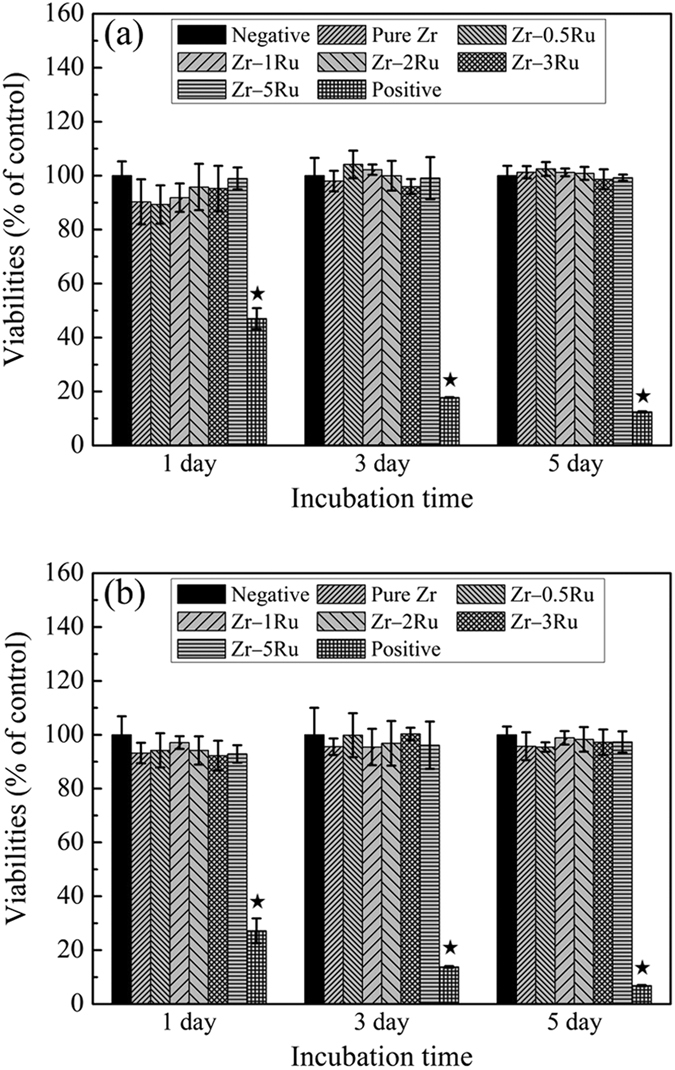
Cell viabilities after culturing in pure Zr and Zr–Ru alloys’ extraction media for 1, 3 and 5 days: (**a**) L-929 cell line and (**b**) MG 63 cell line (^⋆^indicating p < 0.001 when comparing with negative control).

Figure 6 illustrates the L929 (Fig. 6(a)) and MG63 (Fig. 6(b)) proliferations after culturing in extraction media of pure Zr and Zr–Ru alloys for different time periods. It could be seen that, after 1, 3 and 5 days of culture, the cell viabilities of pure Zr and Zr–Ru alloys were almost the same as that of the negative group, and the statistic analysis indicated no significant difference among negative control, pure Zr and Zr–1Ru alloy groups (p > 0.05). Moreover, the cell morphology of the pure Zr and Zr–Ru alloy groups are similar to that of the negative control group, i.e. healthy, well spreading and stretching, spindle-shaped or cellular polygon-shaped, converging and laminipodia could be observed (Supplementary Information S5).

**Figure 7:**
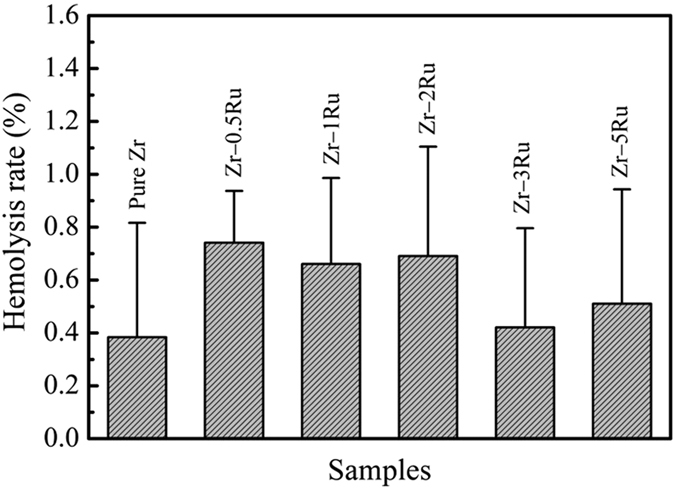
Hemolysis rate of pure Zr and Zr–Ru alloys.

Figure 7 shows the hemolysis rate of pure Zr and Zr–Ru alloys. The hemolysis rates of pure Zr and Zr–Ru alloys are quite low (less than 1%), much lower than 5%, the safe value for biomaterials according to ISO 10993-4 standard, indicating that the pure Zr and Zr–Ru alloys could not cause destructive effect on erythrocyte or hemolysis when contacted with blood.

### Magnetic susceptibility of Zr–Ru alloys

**Figure 8:**
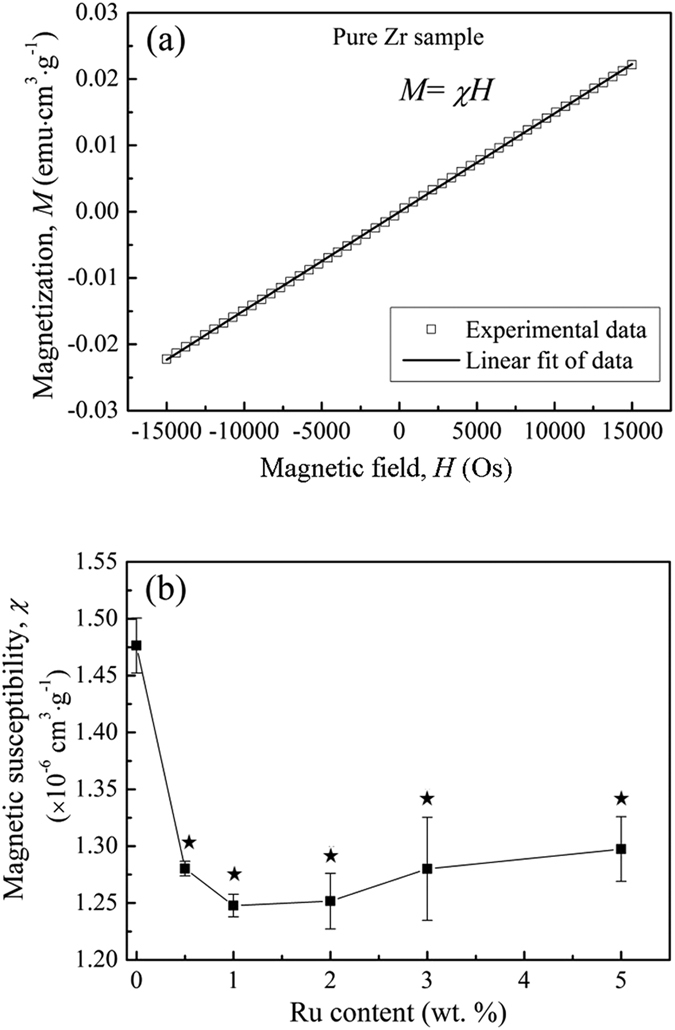
(**a**) The magnetization of pure Zr as function of applied magnetic field at room temperature and the corresponding linear fit, and (**b**) magnetic susceptibility variations of pure Zr and Zr–Ru alloys(^⋆^indicating p < 0.001 when comparing with pure Zr).

The magnetization variation of pure Zr vs. the applied magnetic field at room temperature is shown in Fig. 8(a). Similar to pure Zr, the Zr–Ru alloys showed uniformly a linear variation relationship between magnetization and applied magnetic field, therefore the curves are omitted here. Instead, the magnetic susceptibilities of pure Zr and the Zr–Ru alloys, determined by the slope through linear fitting of the data, are plotted in Fig. 8 (b). It is obviously seen that after adding the alloying element Ru, the magnetic susceptibility significantly decreased. The mostly effective composition point lies in 1% Ru addition, with the magnetic susceptibility being the lowest, 1.247 × 10^−6^ cm^3^·g^−1^, indicating much better MRI compatibility compared with pure Zr (the magnetic susceptibility is 1.475 × 10^−6^ cm^3^·g^−1^).

## Discussion

In our recent work^18^, binary Zr–1X (X = Ti, Nb, Mo, Cu, Au, Pd, Ag, Ru, Hf and Bi) alloy models were used to screen the best alloying element to Zr exhibiting excellent biocompatibility and MRI compatibility, and element Ru was found to be the most effective element. Here in the present work, we tried to optimize the best addition content for element Ru using Zr–Ru binary alloy models with various Ru content (0.5 wt.%, 1 wt.%, 2 wt.%, 3 wt.%, 5 wt.% and 10 wt.%), and based on the experimental results, it can be seen that:

- (1) *For the mechanical property consideration, the Ru content should be lower than 2* *wt.% for Zr*–*Ru alloys*; With the addition of Ru alloy element, both the strength and hardness of Zr–Ru alloys were significantly enhanced compared with that of pure Zr. But for the Zr–Ru alloys containing higher Ru content (higher than 2 wt.%), a significant elongation decrease was observed, which might be attributed to the appearance of ω phase and the RuZr precipitate, which generally bring about destructive effect on the ductility and toughness of biomedical alloys as biocompatible materials and therapeutic devices^19^.
- (2) *For the corrosion resistance consideration, Zr*–*Ru alloys with the Ru content(0.5~5* *wt.%) are better than pure Zr*; When alloying Ru element, the corrosion resistance of Zr increased significantly, which can be proved by the corrosion potential (*E*_*corr*_) and the breakdown potentials (*E*_*tran*_) shift in the noble direction over the Ru-free pure Zr control group. The present result suggests Ru addition can confer a greater resistance to corrosion in Zr-based alloys, reducing both the propensity for the alloy to act as an anodic site and the transient current for voltages up to +1.1 V above *E*_*corr*_, supportive of the ability of Ru addition to Zr-based alloys in improving resistance to corrosion.
- (3) *For the cytocompatibiity and hemocompatibility consideration, Zr*–*Ru alloys with the Ru content(0.5~5* *wt.%) are as good as pure Zr*; Similar to the alloying elements Nb, Mo, Rh, Pd and Ag, Both elements Zr and Ru belong to the 4*d* transition metals in the periodic table, and they are extremely biocompatible, exhibiting low ionic cytotoxicity *in vitro*, excellent biocompatibility *in vivo*, no evidence of mutagenicity or carcinogenicity, and osteocompatibility equaling or exceeding that of the conventional pure Ti and Ti-based biomedical materials and therapeutic devices^9^,^10^,^11^,^12^,^13^,^14^.
- (4) *For the MRI compatibility consideration, Zr*–*Ru alloy with the Ru content of 1* *wt.% shows the lowest magnetic susceptibility value*; It is well known that MRI is a non-invasive diagnostic tool that does not pose the danger of exposing patients to ionizing radiation, and it avoids using nephrotoxic contrast agents^20^. However, under a magnetic field with intense strength, paramagnetic metals with high magnetic susceptibility (χ), such as biomedical 316L stainless steel, Co–Cr alloys and Ti-based alloys can generate artifacts in the images as a result of the distortion of the magnetic field^21^. Thus, in regard to decrease the artifacts and get the authentic diagnostic MRI imaging, developing novel metallic implant with an ultralow magnetic susceptibility, thus having better MRI compatibility is of considerable interest. The present work demonstrated that the ultralow magnetic susceptibilities of the Zr–Ru alloys (1.25 × 10^−6^ cm^3^·g^−1^–1.29 × 10^−6^ cm^3^·g^−1^ for Zr–Ru alloys) is only one-third that of Ti-based alloys (Ti–6Al–4V, ~3.5 × 10^−6^ cm^3^·g^−1^, CP Ti and Ti–6Al–7Nb, ~3.0 × 10^−6^ cm^3^·g^−1^), and one-sixth that of Co–Cr alloys (Co–Cr–Mo, ~7.7 × 10^−6^ cm^3^·g^−1^)^19^, which are the well-known commonly widely used for biomedical materials and therapeutic devices with high magnetic susceptibility, unsuitable for MRI diagnostics.

It is well known that in the current clinical therapies and operations, especially for minimally invasive surgeries and therapies, certain interventional medical implants and devices, such as catheters, filters, wire guides, stent grafts, needles, and the like, may need MRI procedures to determine the position of the device and deliver drugs during procedures viewed with MRI techniques. Among the Zr–Ru alloy series, Zr–1Ru demonstrates enhanced mechanical properties, excellent corrosion resistance and cell viability with lowest magnetic susceptibility, and thus is the optimal Zr–Ru alloy system as above mentioned biomedical materials and therapeutic devices used under MRI techniques and diagnostics environments.

## Methods

### Alloys preparation

The binary Zr–Ru alloys with the nominal chemical composition of 0.5%, 1%, 2%, 3%, 5% and 10% (in weight percentage, wt.%) were prepared in a non-consumable arc melting furnace under an Ar atmosphere. Each alloy ingot was re-melted six times by inversion to make sure its chemical homogeneity. The actual chemical compositions of resulting Zr–Ru alloys were determined by energy dispersive spectrometry (EDS), as given in Supplementary Information S6. Some samples were cut directly by electro-discharge machining from the as-cast ingots, and were named after “as-cast” samples. Part of obtained ingots were further hot-rolled to 3 mm thick sheets at 800 °C, and then cold-rolled into thin plates of 1.5 mm in thickness by a total reduction of 50%. The test samples were cut by electro-discharge machining from the as-rolled plates and then annealed at 600 °C for 2 hours (hereafter noted as “annealed” samples).

### Microstructural characterization

X-ray diffractometer (XRD, Philips X′Pert Pro, Holland) with a Ni filtered Cu K*α* radiation was employed to analyze the phase constitution of the experimental Zr–Ru alloys.

The microstructure of these alloys was examined using an optical microscopy (OM, BX51M Olympus, Japan). The specimens were mechanically polished via a standard metallographic procedure and then etched in a solution of HF, HNO_3_ and H_2_O, with the volume ratio of 10%: 45%: 45%, respectively.

### Mechanical properties tests

The uniaxial tensile test was performed with an initial strain rate of 5 × 10^−4^ s^−1^ on a mechanical tester (Instron5969, USA) at room temperature. The strip specimens with dimension of 50 mm × 3 mm × 1 mm were prepared, using the central 20 mm as gage length. The 0.2% offset yield strength (YS) and the ultimate tensile strength (UTS) were obtained from the stress-strain curve. For each alloy, five duplicate specimens were tested. The tensile fracture surfaces were further observed by scanning electron microscopy (SEM, Hitachi S-4800, Japan).

Hardness of Zr–Ru alloys was determined by a digital Vickers microhardness tester (HMV-2T, Shimadzu, Japan) with a 1.961 N load and 15 s dwell time. Six points were chosen and measured in different positions of each sample to get an average value.

### Electrochemical corrosion measurement

The electrochemical experiments were conducted in a three-electrode system at a constant temperature of 37.0 ± 0.5 °C in a water bath. A saturated calomel electrode (SCE) and a palladium foil were used as reference electrode and counter electrode, respectively. The electrolyte was Hank’s simulated body fluid with pH = 7.4. Prior to testing, the samples were wet-ground to 2000 grit, and were later cleaned in acetone, ethanol and de-ionized water in an ultrasonic bath. The open-circuit potential (OCP) of each sample was continuously monitored for 2 hours in Hank’s solution. At the end of the exposure of 2 hours, the potentiodynamic polarization measurement was conducted from −0.8 V to 1.5 V (vs. SCE) for each sample, with the scan rate being 1 mV/s. The surface morphology after corrosion test were studied by scanning electron microscope (SEM, Hitachi S-4800, Japan) observation. X-ray photoelectron spectroscopy (XPS) analysis (Axis Ultra, Kratos Analytical Ltd.) was performed to analyze the surface composition of the passive films developed on the experimental alloys’ surface. The test conditions are as follows: mono Al Kα (1486.6 eV) radiation at vacuum pressure of 10^−9^ bar, 15 kV and 15 mA. The binding energy was calibrated using Cls hydrocarbon peak at 284.8 eV.

### Cell experiments

MG 63 osteoblast-like cells and L-929 fibroblast cells were used for cytocompatibility test in this study. Cells were cultured in minimum essential medium (MEM), containing 10% fetal bovine serum (FBS), 100 U/ml penicillin and 100 μg/ml streptomycin. They were incubated in a humidified atmosphere with 5% CO_2_ at 37 °C in a cell incubator. The culturing medium was changed for every three days throughout cell experiments. When the cells grew to confluence, they were detached using 0.25% trypsin and collected in the fresh medium for the subsequent cell experiments.

The cell viability of experimental Zr–Ru alloy groups were evaluated by an indirect method according to the instruction of ISO 10993-12:2007. Cell culture medium (MEM) was used as a negative control and MEM containing 10% dimethylsulfoxide (DMSO) as a positive control. Extracts of testing materials were obtained using serum free MEM as the extraction medium. For 3 cm^2^ of material, 1 ml medium was used and extraction was conducted for 72 hours at 37 °C. Cells were seeded in 96-well plates at a density of 4 × 10^3^cells/100 μl medium and incubated for 24 hours to allow cell attachment. Then culture mediums were substituted by the extracts obtained from the studied materials, and incubated for 1, 3 and 5 days, respectively. Within each of the culturing period, the cell morphology was observed under inverted phase contrast microscope. After the cell morphology observation, 10 μl 3-(4, 5-Dimethylthiazol-2-yl)-2, 5-diphenyltetrazolium bromide (MTT, 5 mg/ml) was placed in each well and the well plates with MTT were incubated for 4 hours in darkness. After that, 100 μl formazan solubilization solution (10% sodium dodecyl sulfate (SDS) in 0.01 M HCl) was added in each well overnight in the incubator. The spectrophotometrical absorbance of the product in each well was measured by microplate reader (Bio-RAD680) at 570 nm with a reference wavelength of 630 nm.

### Hemolysis rate test

Healthy human blood from a volunteer containing sodium citrate (3.8 wt.%) in the ratio of 9:1 was taken and diluted with normal saline (4:5 ratio by volume). Samples were dipped into a standard tube containing 10 ml of normal saline that were previously incubated at 37 °C for 30 min. Then 0.2 ml of diluted blood was added to this standard tube and the mixtures were incubated for 60 min at 37 °C. Similarly, normal saline solution was used as a negative control and deionized water as a positive control. After this period, all the tubes were centrifuged for 5 min at 3000 rpm and the supernatant was carefully removed and transferred to the cuvette for spectroscopic analysis at 545 nm using microplate reader (Bio-RAD680).

The methods were carried out in accordance with the approved guidelines. All experimental protocols were approved by the Institutional Ethics Committee of Peking University. Written informed consent was obtained from all subjects.

### Magnetic susceptibility (χ ) measurement

The magnetic properties of experimental Zr–Ru alloys were investigated using a SQUID-VSM (Superconducting Quantum Interference Device-Vibrating Sample Magnetometer, Quantum Design, USA) at room temperature. The magnetization (*M*) of sample as a function of applied magnetic field (*H*) was measured and recorded, and its magnetic susceptibility, *χ = M/H*, was obtained from the slope through linear fitting of the data. The applied magnetic field (*H*) was set from -15000 Os to + 15000 Os. For each experimental group, three duplicate specimens were prepared and tested.

### Statistical analysis

Statistical analysis was performed with SPSS 18.0 software. Differences between groups were analyzed using one-way ANOVA, followed by post-hoc Tukey’s test. Differences are considered statistically significant at a value of p < 0.05 (denoted as *); very significant at a value of p < 0.01(denoted as ^#^) and extremely significant at a value of p < 0.001 (denoted as^⋆^).

## Additional Information

**How to cite this article**: Li, H.F. *et al.* Design and development of novel MRI compatible zirconium-ruthenium alloys with ultralow magnetic susceptibility. *Sci. Rep.* **6**, 24414; doi: 10.1038/srep24414 (2016).

## Supplementary Material

Supplementary Information
