# Supplementary material for: Design and development of novel MRI compatible zirconium- ruthenium alloys with ultralow magnetic susceptibility
Source: Sci Rep. 2016 Apr 19;6:24414. doi: 10.1038/srep24414 (PMC4836298; doi:10.1038/srep24414)
Supplement: Supplementary Information [file srep24414-s1.doc]

**Supplementary Information**

**Design and development of novel MRI compatible zirconium-** **ruthenium alloys with ultralow magnetic susceptibility**

H.F. Li, F.Y. Zhou, L. Li, Y.F. Zheng


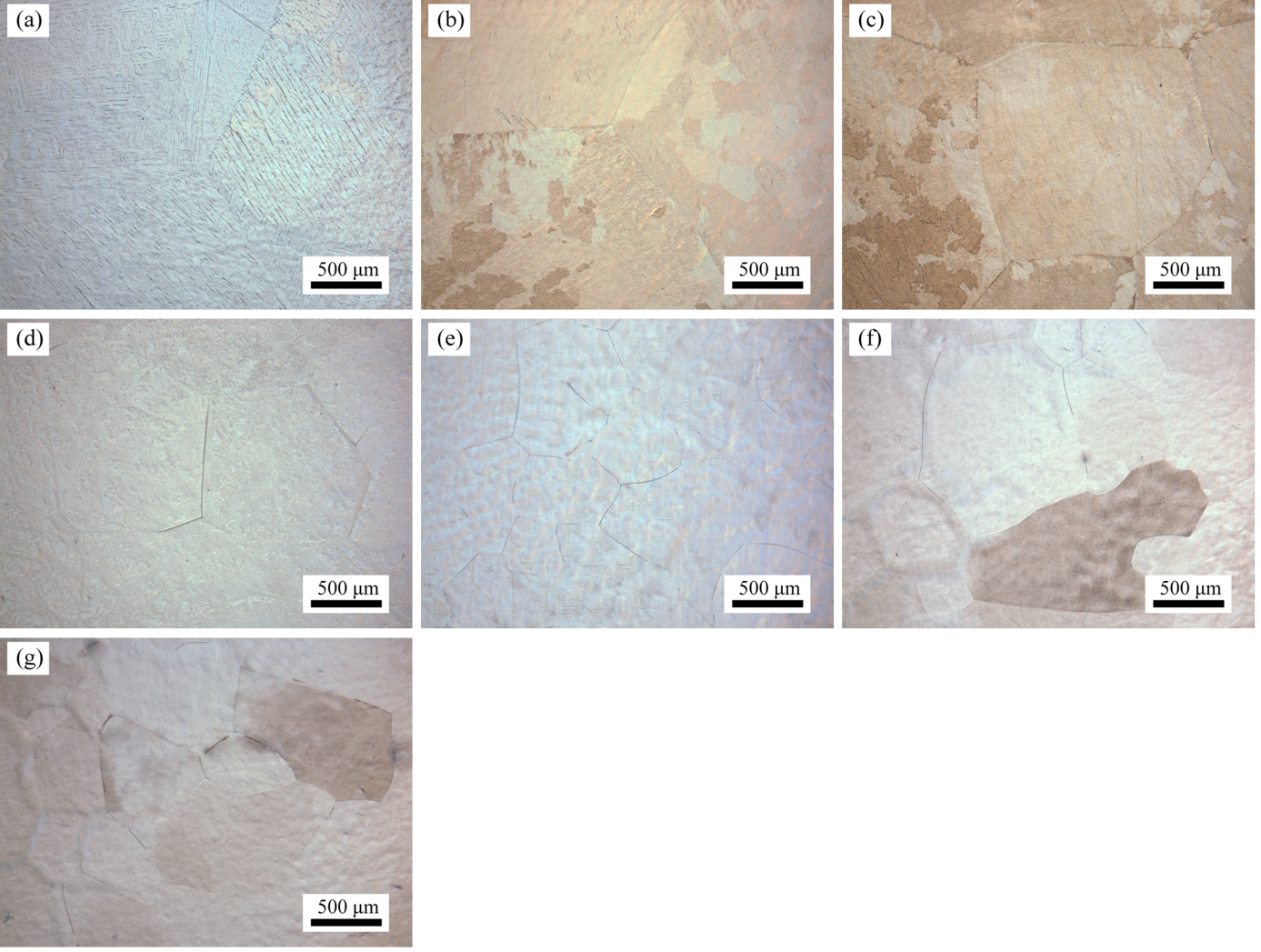


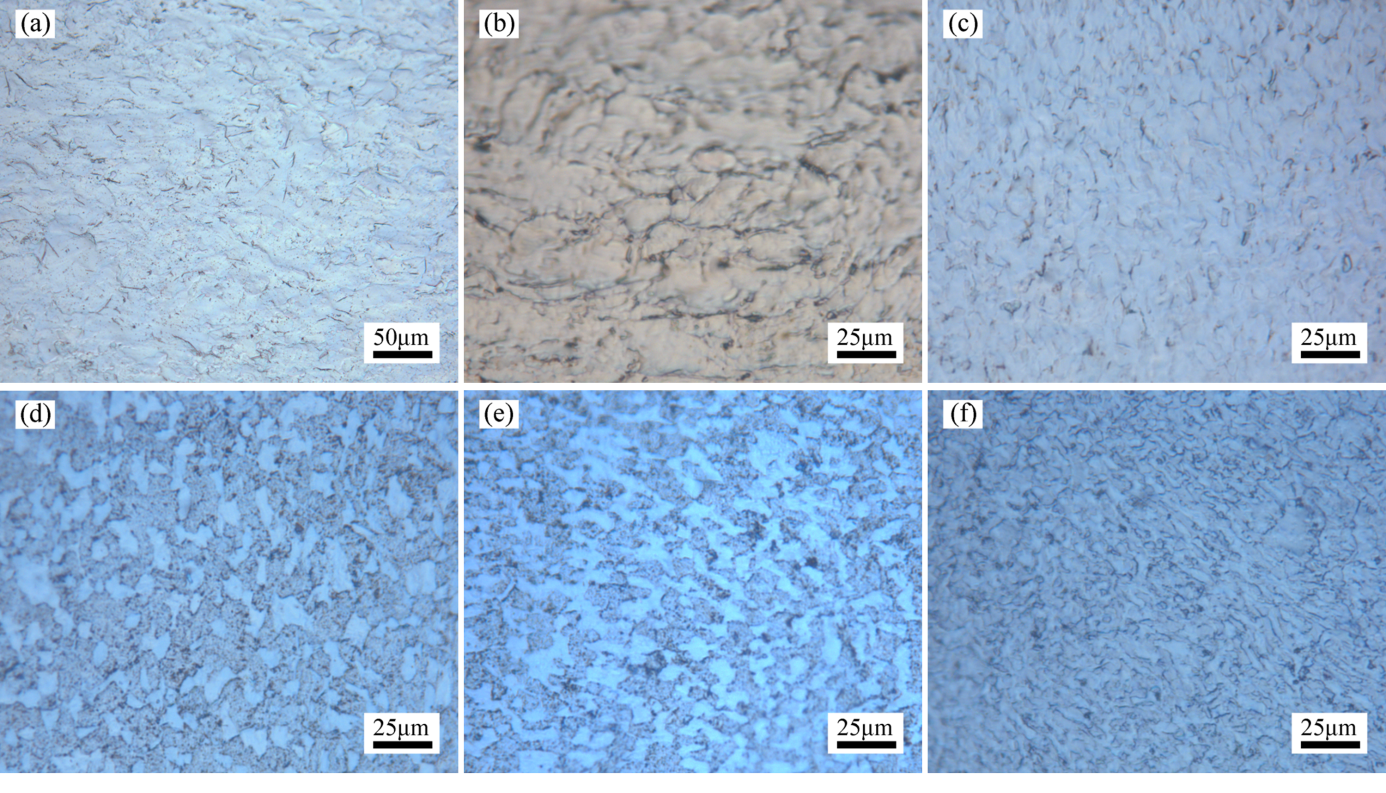


S1. Optical micrographs of as-cast (A) and annealed (B) pure Zr and Zr−Ru alloys: (a) pure Zr, (b) Zr−0.5Ru, (c) Zr−1Ru, (d) Zr−2Ru, (e) Zr−3Ru, (f) Zr−5Ru and (g) Zr−10Ru alloys

S2. Corrosion property parameters of pure Zr and Zr−Ru alloys in Hank's solution

| Material | OCP (V) | *E*_corr_ (V) | *i*_corr_ (10^-7^ A/cm^2^) | *E*_tran_ (V) |
| --- | --- | --- | --- | --- |
| Pure Zr | -0.681±0.039 | -0.688±0.043 | 1.125±0.223 | 0.426±0.118 |
| Zr−0.5Ru alloy | -0.648±0.029 | -0.651±0.034 | 1.149±0.502 | 0.568±0.026 |
| Zr−1Ru alloy | -0.638±0.044 | -0.639±0.049 | 1.112±0.430 | 0.577±0.093 |
| Zr−2Ru alloy | -0.604±0.013 | -0.605±0.015 | 1.068±0.231) | 0.571±0.048 |
| Zr−3Ru alloy | -0.573±0.014 | -0.585±0.037 | 1.082±0.042 | 0.624±0.089 |
| Zr−5Ru alloy | -0.533±0.018 | -0.532±0.021 | 0.977±0.168 | 0.573±0.107 |


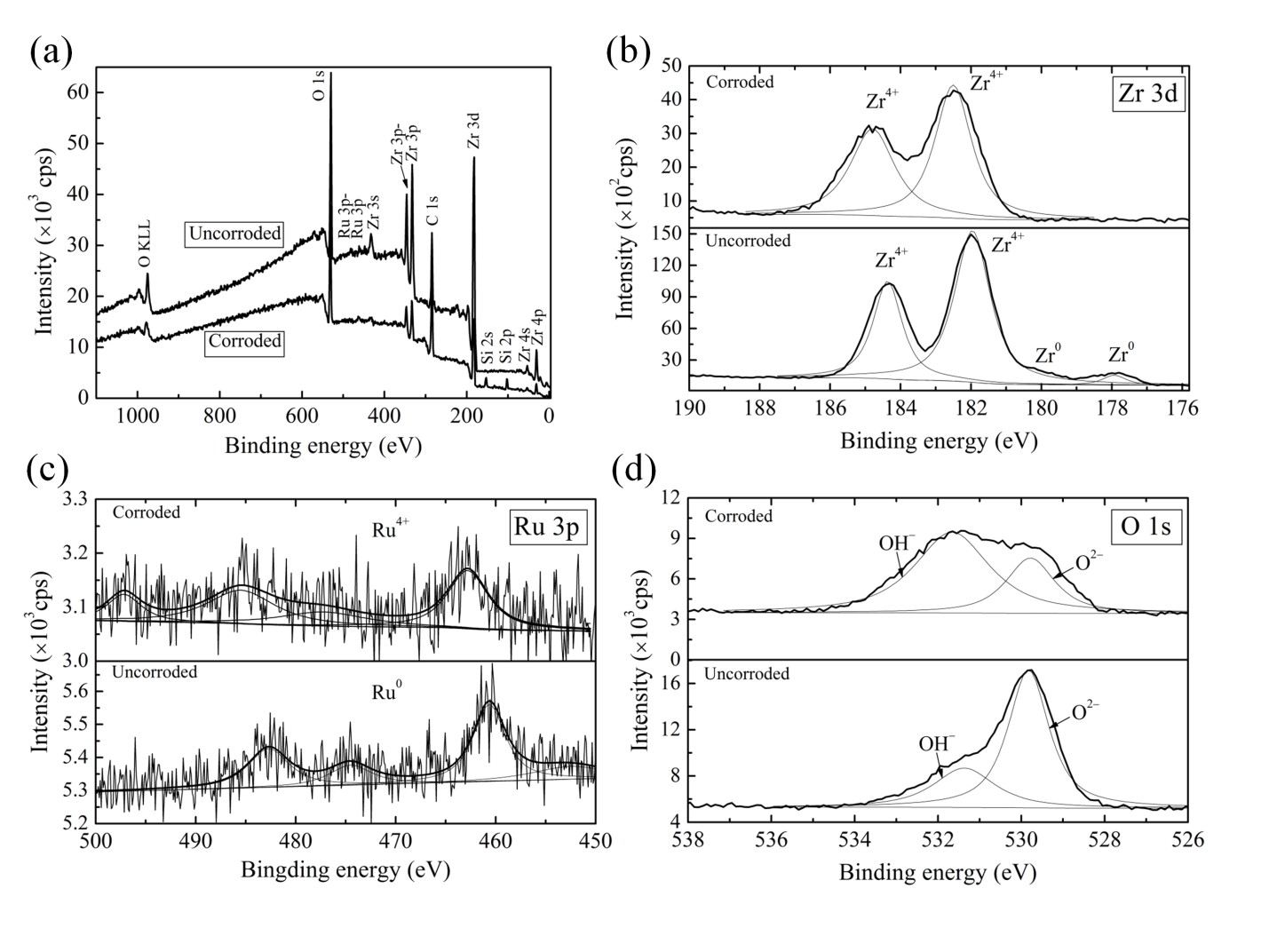


S3. XPS survey spectra (a) and high solution XPS spectra of (b) Zr 3d, (c) Ru 3p and (d) O 1s regions obtained from uncorroded and corroded Zr−5Ru alloy sample surface

S4. The element distributions of Zr−Ru alloys after corrosion test detected by XPS analysis

| Alloys | Content (mol %) | | |
| --- | --- | --- | --- |
|  | Zr | Ru | O |
| Zr−0.5Ru | 27.45 | − | 72.55 |
| Zr−1Ru | 27.56 | − | 72.43 |
| Zr−2Ru | 28.79 | 0.42 | 70.79 |
| Zr−3Ru | 28.43 | 0.60 | 70.97 |
| Zr−5Ru | 27.28 | 0.86 | 71.86 |


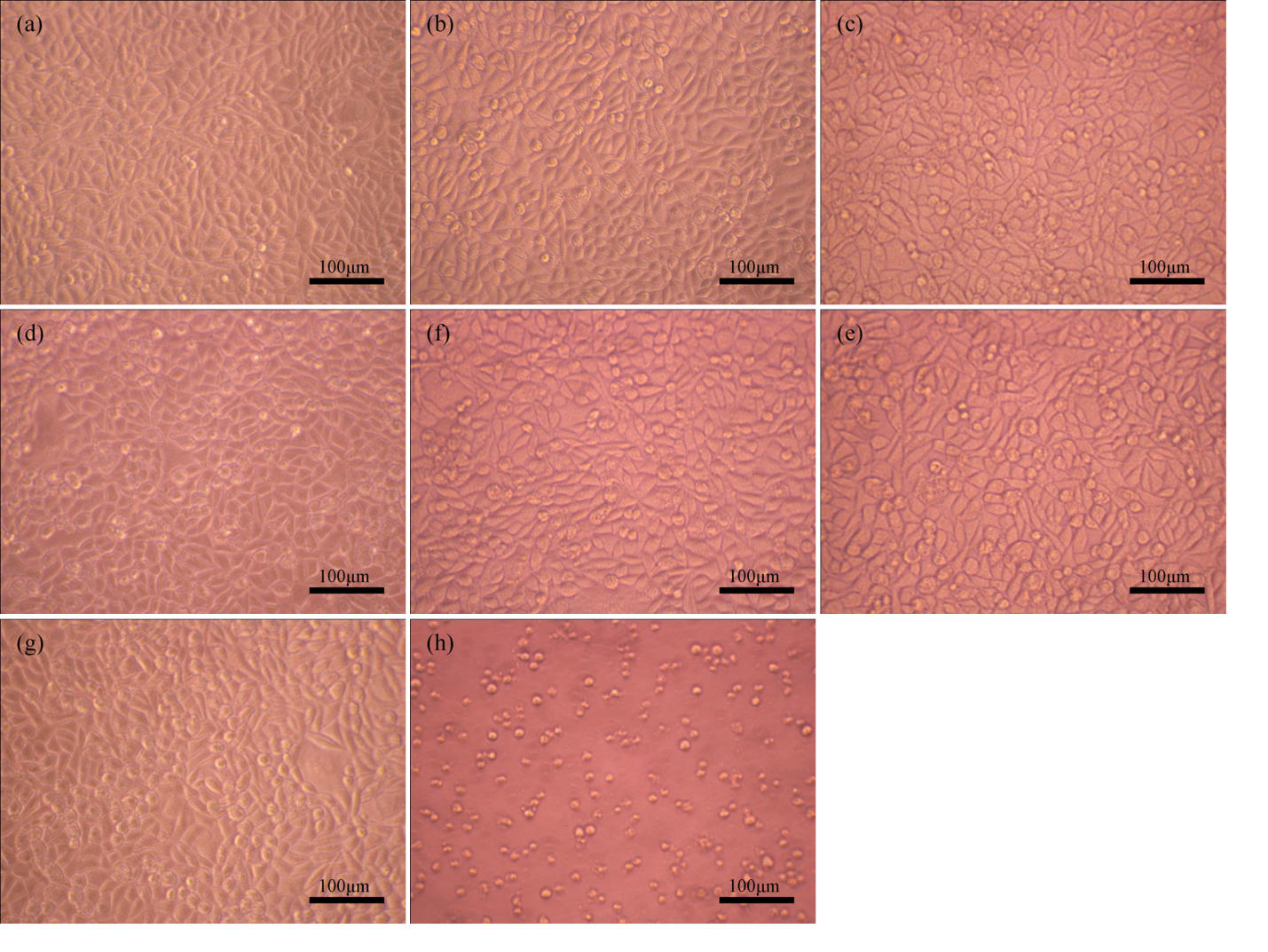


S5. Morphological observations of L-929 cells after culturing in pure Zr and Zr−Ru alloys' extracts for 5 days: (a) negative control, (b) pure Zr, (c) Zr−0.5Ru, (d) Zr−1Ru, (e) Zr−2Ru,

(f) Zr−3Ru, (g) Zr−5Ru, and (h) positive control

S6 Chemical composition (wt. %) of Zr−Ru alloys detected by EDS analysis

| Alloy | Zr−0.5Ru | Zr−1Ru | Zr−2Ru | Zr−3Ru | Zr−5Ru | Zr−10Ru |
| --- | --- | --- | --- | --- | --- | --- |
| Ru content | 0.722±0.045 | 1.277±0.083 | 2.160±0.147 | 2.915±0.086 | 4.383±0.437 | 9.497±1.014 |
